# Small temporal asynchronies between the two eyes in binocular reading: Crosslinguistic data and the implications for ocular prevalence

**DOI:** 10.3758/s13414-021-02286-1

**Published:** 2021-05-27

**Authors:** Ruomeng Zhu, Mateo Obregón, Hamutal Kreiner, Richard Shillcock

**Affiliations:** 1 School of Philosophy, Psychology and Language Sciences, University of Edinburgh, 3 Charles St, Edinburgh, EH8 9AD UK; 2 School of Informatics, University of Edinburgh, Edinburgh, UK; 3 Behavioural Sciences, Ruppin Academic Center, Hadera, Israel

**Keywords:** Binocular reading, Eye-tracking, Ocular prevalence, English, Chinese

## Abstract

We investigated small temporal nonalignments between the two eyes’ fixations in the reading of English and Chinese. We define nine different patterns of asynchrony and report their spatial distribution across the screen of text. We interpret them in terms of their implications for ocular prevalence—prioritizing the input from one eye over the input from the other eye in higher perception/cognition, even when binocular fusion has occurred. The data are strikingly similar across the two very different orthographies. Asynchronies, in which one eye begins the fixation earlier and/or ends it later, occur most frequently in the hemifield corresponding to that eye. We propose that such small asynchronies cue higher processing to prioritize the input from that eye, during and after binocular fusion.

Binocular coordination during reading has become an important theme in reading research in the past 2 decades (e.g., Liversedge et al., 2006; Shillcock et al., 2010); how do the two eyes coordinate their efforts? At the same time, researchers have been increasingly interested to compare reading behaviors in different languages and their orthographies (e.g., Hsiao et al., 2018; Liversedge et al., 2016); how much is language-specific, how much is universal? We assume that readers coordinate their eyes to optimize higher visual processing. In reading, binocular fusion is crucial to this process and must involve deciding how much higher visual processing should rely on the input specific to each eye (Ehrenstein et al., 2005). This “ocular prevalence” has typically been discussed in terms of quantitatively resolving stereo-disparate images (cf. Heinrich et al., 2005; Kommerell et al., 2003; Kromeier et al, 2006) but is a wider, subtler issue of combining similar inputs (Shillcock et al., 2010). How is this fluid division of labor between the eyes mediated?

Chinese orthography is considered logographic, or ideographic. It is visually denser than English (cf. Hsiao et al., 2018). Its greater informational density elicits smaller saccades and longer fixations (cf. Hsiao, 2017; Liversedge et al., 2016), compared with English. Ho and Bryant (1999) suggest learning to read English and Chinese requires different visual skills. Similarly, McBride-Chang et al. (2015) propose that reading Chinese co-opts more regions of the brain, compared with alphabetic texts (see, also, Tan et al., 2005). Despite such processing differences, researchers have also proposed commonalities—or universals (e.g., Feng et al., 2009). Sun et al. (1985) reported similar patterns of saccades and fixations in the two orthographies. Liversedge et al. (2016) reported putative universal effects concerning word frequency, word length, and word predictability across English, Chinese, and Finnish.

Cross-linguistic research on binocular coordination in Chinese and in English has mainly been concerned with the *spatial* characteristics of eye movements and with binocular fixation disparities during reading (e.g., Hsiao et al., 2018; Kirkby et al., 2008; Liversedge et al., 2006; Shillcock et al., 2010), specifying the location of the two eyes’ fixations. Hsiao (2017) investigated spatial binocular disparities with *temporally conjugate* fixations (i.e., fixations in which the right and left eye both start and end their fixations at the same time.) She reports (chiefly quantitative) similarities and differences across the two languages.

However, *temporal disjugacy*, the small-scale temporary unyoking of the two eyes’ fixations in time, has been less explored. Below, we report binocular fixation data in reading English and Chinese in terms of small temporal asynchronies between the two eyes during multiline reading. We explore nonalignments in the timing of the start and end points of the fixation of the two eyes.

There are viewing conditions and visual tasks, such as sighting and pointing tasks, in which the viewer can only engage accurately with the real world by allowing one eye’s input to dominate completely (Kommerell et al., 2003; Porac & Coren, 1976). Such *ocular dominance* is skewed toward the right eye, across the population (Hillemanns, 1927). The implications of ocular dominance are unclear; Mapp et al. (2003) have claimed that the dominant eye may have “no unique functional role in vision” (p. 310).

However, there are more pervasive viewing conditions and tasks in which the inputs from the two eyes can be combined. The contradiction between the inputs from the two eyes gives rise to depth perception, essential in everyday tasks of fine manual control, such as threading a needle. One eye’s input can still be prioritized in the final conscious percept—*ocular prevalence*. Stereo-disparate images have primarily been used in the operational definition and exploration of ocular prevalence, in which Kommerell et al. (2003) find that the right and left eyes are equally likely to be preferred in different individuals. Crucially, Kromeier et al. (2002) show that ocular prevalence is still compatible with high stereo acuity at small stereo disparities.

What we consider below is the class of differences between the inputs from the two eyes in which there is no resulting depth perception, because there is no such stereo-disparity in the text stimulus (cf. Shillcock et al., 2010). Instead, the differences between the two inputs may result from viewing angle, as when the two eyes fixate the left end of a line of text; the left eye will be closer, the right eye’s view is more skewed, for instance. The differences in input may also result from inherent anatomical differences, such as size and shape of the eyeball. These differences in inputs still present a computational challenge to fuse the inputs to best advantage, and there is still scope for ocular prevalence (the “graded quantification of the balance between the eyes”; Kommerell et al., 2003, p. 1397) to improve the division of labor between the two eyes. Such a quantitative, graded difference in cortical activation has been reported for dominance (Rombouts et al., 1996), at least partly grounded in anatomy and physiology.

Below, we are concerned with how the conditions for ocular prevalence may play out in sustained reading from a screen. We tested two hypotheses:

- *Hypothesis (1):* The left eye will tend to begin fixating earlier and stay fixating longer in the left visual field, and the right eye will be similarly prioritized in the right visual field, thereby facilitating the appropriate switching of ocular prevalence.

This hypothesis is based on our prediction of a division of labor between the two eyes. There are differences in the projection of each eye to the primary visual cortex, favoring contralateral projection from the eye nearest the hemifield (e.g., left eye and left visual field; Toosy et al., 2001; see Discussion, below). For eye-specific effects involving spatial attention, see, for instance, McCourt et al. (2001).

- *Hypothesis (2):* A very similar pattern of binocular temporal asynchronies will be found even across the two very different orthographies of English and Chinese.

This null hypothesis is based on our review, above, of some of the claims regarding similar and different reading behaviors in Chinese and English. If we find crosslinguistic dissimilarities in timing asynchronies, this will imply that higher cognitive processing, perhaps specific to reading, is involved. If we find very similar qualitative patterns, it will imply that peripheral, anatomically based processing underlies the timing asynchronies.

## Method

### Participants

We paid 36 Chinese and 38 English native speakers for their participation.^1^ All tested and reported as having normal or corrected-to-normal vision. They were students at the University of Edinburgh. The Chinese participants all had English as a second language. The English participants had a variety of exposures to other languages. We do not explore the role of demographic variables below.

### Apparatus

Participants sat in a room with diffused lighting, and watched a 22-in. Ilyama Vision Master Pro 514 display, at a distance of 75 cm. The screen resolution was 1,024 × 768 pixels. A chin rest and forehead support kept the head stable. The eye tracker was an SR Research EyeLink II head-mounted video-based tracker**.**

### Stimulus materials and procedure

We recorded eye movements binocularly, using the EyeLink II’s pupil tracking and corneal reflection, and sampled at 500 Hz (i.e., every 2 ms), during the reading of English (24-pt monospaced Monaco font) and Chinese (PMingLiU, standard print) texts, each comprising 21 newspaper stories,^2^ with a total of 5,000 words for each language, presented in black characters on a light background, on consecutive pages with up to five left-justified lines of text each. One monospaced English letter occupied 14.4 pixels; one Chinese character occupied 28 pixels. The stimuli were intended to be comparable between languages, in form and content, based on the intuitions of native speakers. The maximum line length corresponded to 64 English characters. The text was left-justified in both orthographies; note that the nature of Chinese orthography means that line length is much more regular for Chinese, compared with a ragged right edge for English, meaning that English lines were on average shorter. Readers were calibrated monocularly with a 9-point fixation grid while occluding the other eye with a black paper shade. Participants fixated a black fixation disc before each page of text was displayed and fixated a square at the bottom right of each page after finishing reading it. They responded on the keyboard to a yes/no question after each story, to ensure reading for meaning. Mean comprehension accuracy was 77% and 82% for English and Chinese, respectively, indicating that participants read for meaning; no data were excluded on this criterion. The grid of fixation targets was presented before the next article, to check the calibration accuracy. The whole recording process consisted of three blocks with intervening rest-breaks, lasting for around 1.5 hours in total.

### Analysis

For each binocular fixation, the start-time offset was calculated as the fixation start time of the right eye minus the fixation start time of the left eye. The end-time offset was calculated analogously. A difference of ±2 ms between events in the two eyes was considered as simultaneous, given the sampling rate. StartTime offset or EndTime offset <2 means the right eye starts or ends earlier than the left eye. StartTime offset or EndTime offset >2 means the left eye starts or ends earlier than the right eye. The asynchronies themselves varied between participants, but trellis graphs (not shown) revealed a robustly similar qualitative pattern across participants. The asynchronies formed a long-tailed distribution beginning in single-digit numbers of milliseconds.

**Fig. 1:**
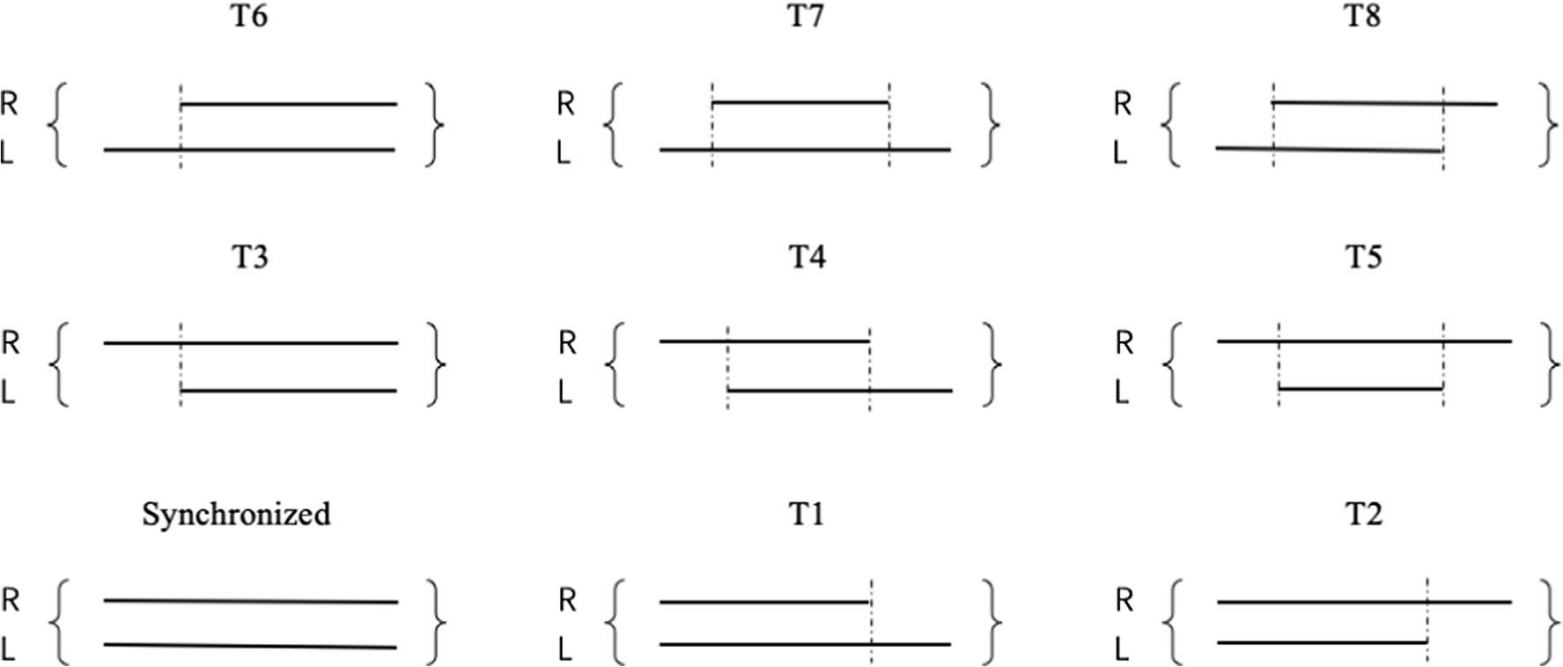
Typology of binocular fixation asynchronies. Left-priority types: T1, T6, T7. Right-priority types: T2, T3, T5 *Note.* Lines are fixation durations

Figure 1 depicts the comprehensive typology of offsets. There are nine possible types of binocular fixation, with different patterns of start-time and end-time offsets. For example, Type 1 shows both eyes starting fixation synchronously and the left eye fixating for longer. We first produced demonstrative graphs to show the distributions of the types on the screen. We then analyzed the data quantitatively with GLMER models, to further characterize the eye-movement behaviors and explore the potential implications for ocular prevalence.

## Results

We analyzed a total of 160,567 binocular fixations (i.e., pairs of individual, temporally overlapping fixations by the left and right eye) for the English readers and 158,794 binocular fixations for the Chinese readers. Below, we first report descriptive statistics from two perspectives: (a) the overall distribution of the different types; (b) the spatial distribution of the types across the screen on which the text stimuli were displayed. Then, we report the quantitative analysis from GLMER models from two perspectives: (a) group (i.e., language, English and Chinese) differences; (b) screen differences under subsets of types (cf. Fig. 1) in each group. The results all together indicate a lawful patterning of binocular behaviors relevant to ocular prevalence, across the visual field.

### Overall results

**Fig. 2:**
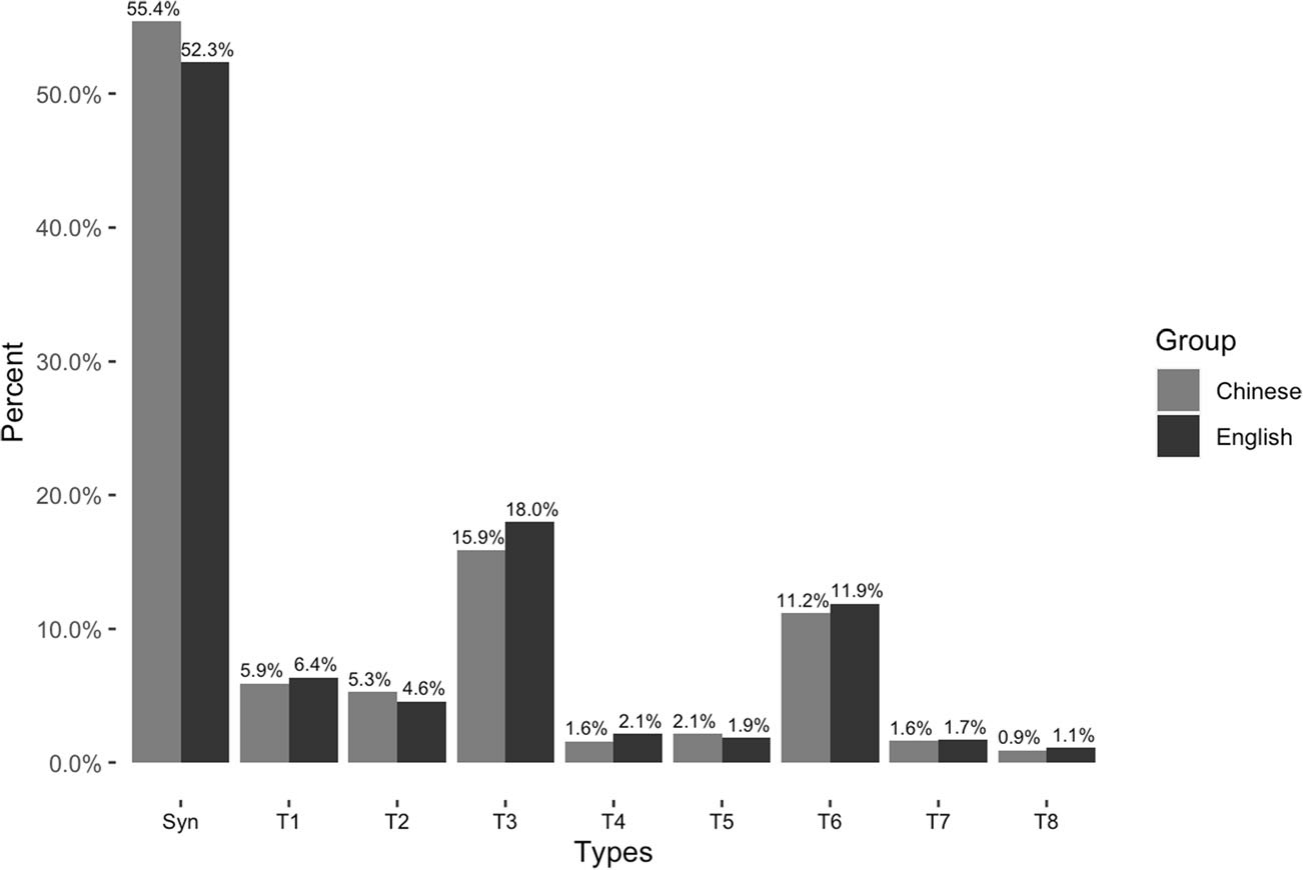
The distribution of types of asynchrony in English and Chinese readers

Figure 2 shows the overall distribution of each type and their percentages among all fixation pairs for English and Chinese readers, respectively. Strikingly similar patterns obtain for both languages, with the three most numerous types of binocular fixation being the synchronized pairs, Type 3 and Type 6; in particular, synchronized binocular fixations account for over half the binocular fixations for both languages. Just over 80% of binocular fixations in both languages end synchronously.

### Spatial distribution

**Fig. 3:**
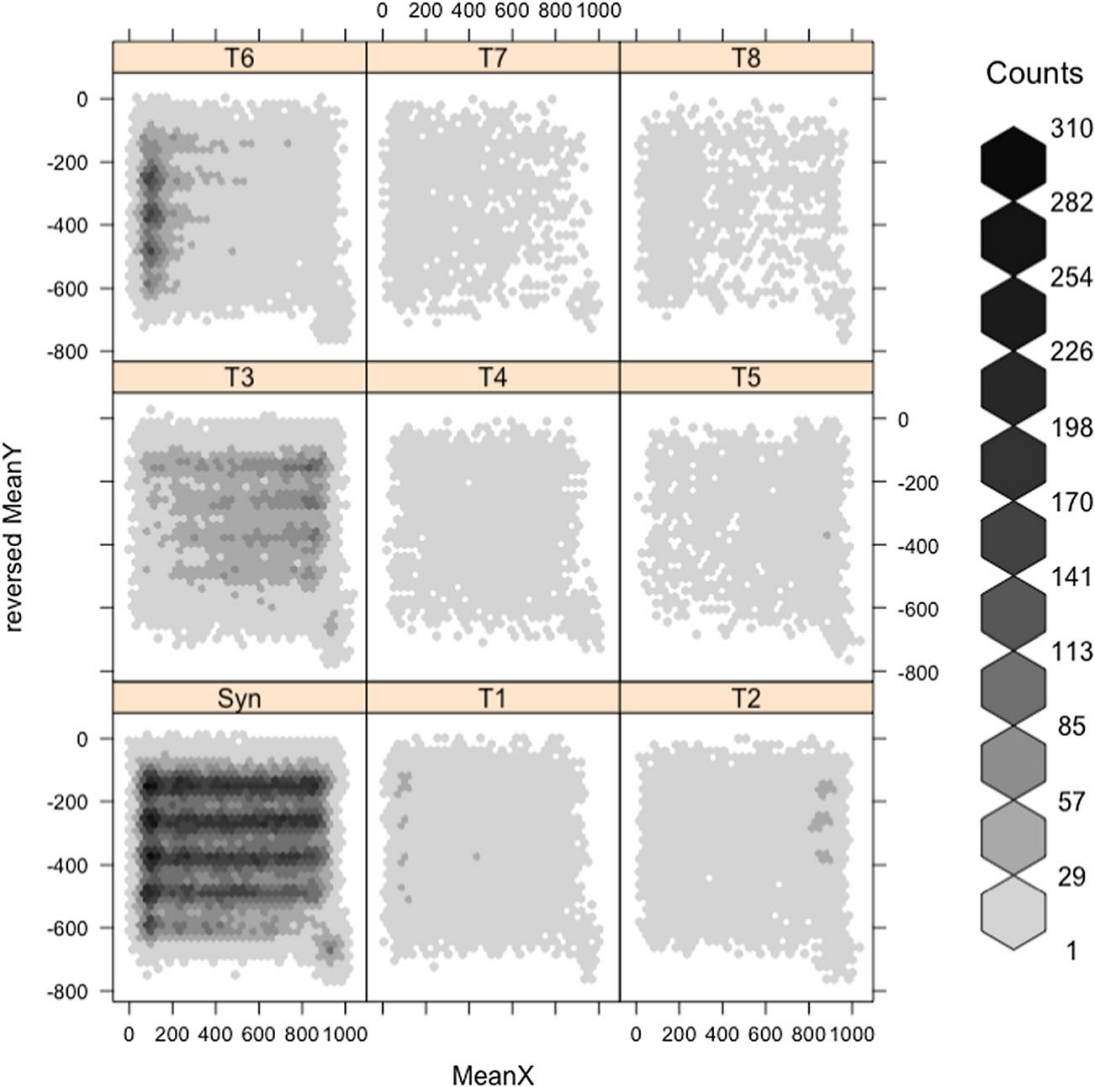
Spatial distribution of Types T1–T8 and synchronous fixations in English readers

Figure 3 shows the spatial distribution of each type on the lines of text, for English readers. The hexbin graph shows the mean coordinates (during the fixation) of the right eye for each binocular fixation, accurately representing the spatial distribution of binocular fixations (the choice of right eye over left has no implications here). The distributions reflect the fact that readers fixated a square at the bottom right of each page after reading it. The legend shows the frequency counts of fixations.

**Fig. 4:**
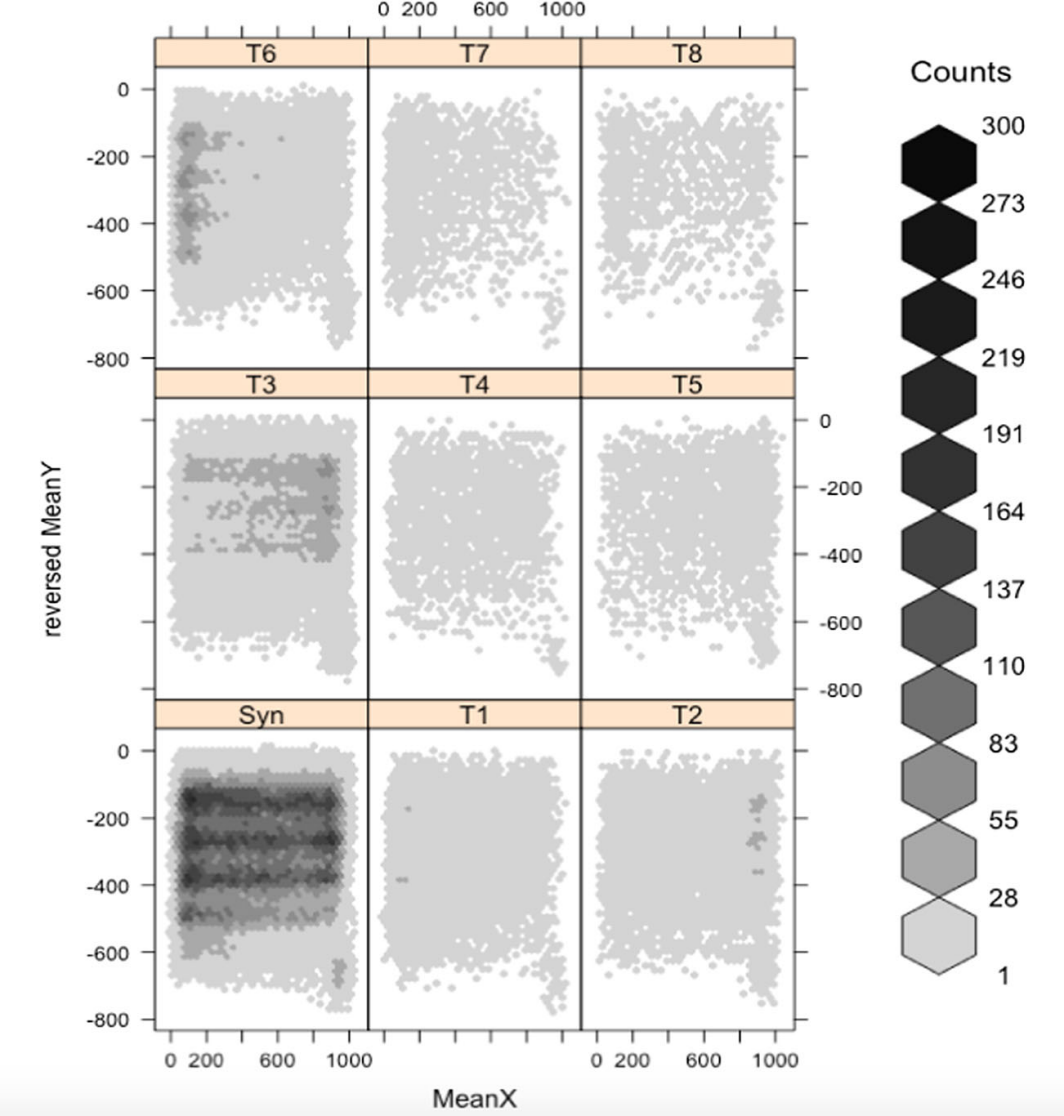
Spatial distribution of Types T1–T8 and synchronous fixations in Chinese readers

The fixation types Syn (synchronized), T3 and T6 show the greatest densities. There are clear differences between left, middle, and end of each line. Syn are concentrated most at the left of lines, but span the whole line. T3 and T6 are skewed to the right and left of lines, respectively. T1 and T2 show slighter skewing to the left and right of lines, respectively. The remaining four types have less skewed distributions. The Chinese (Fig. 4) and English data show qualitatively similar patterns. We divided the screen into left, middle, and right, to investigate the implications for ocular prevalence, in the following analyses.

### Modeling results

We use general linear mixed-effects regression (GLMER) models in the following quantitative analyses, carried out in R using the lme4 software package (Bates et al., 2008). We used the counts of fixation pairs as dependent variables in all the models. As our dependent variable is counts of events, all our modeling used Poisson error distributions. We defined null models with participants, articles, page order (of each article) as random factors; we expected increased predictability through the successive pages of an article. Predictor variables included types (cf. Fig. 1), groups (English, Chinese), the sides of the screen (left, middle, and right, approximating left, middle, and right of lines of text, middle as reference) and tested under subsets of types in each group separately to explore the quantitative distribution of the types. All model fit was assessed using the anova function to compare models. The results show a systematic pattern of binocular behavior across languages, with the Chinese data being somewhat more systematic.

In the first part, we explored our hypotheses within each group separately (i.e., English and Chinese). We explored the effects of synchronized fixations and T1, T2, T3, T5, T6, and T7 asynchronies within each language (i.e., counts of fixations for each comparison). (Types T4 and T8 were omitted from the analyses because their theoretical predictions were ambiguous.) In all of these models, we added sides of the screen (left, middle, right, with the middle as reference) as a predictor variable to the null model.

The majority of the non-Syn types—T1, T2, T3 and T6—are similarly distributed in both groups. The patterns suggest ocular prevalence. First, Table 1 shows that T1 and T6 (left-eye priority) have significantly less value on the right side of the screen compared with the middle (i.e., the reference) in English readers. T6 also has significantly more value on the left side. Similar results can be observed in Chinese readers (see Table 2), particularly in T6; additionally, Chinese T1 has significantly more value on the left side.

**Table 1:** GLMER analysis of screen differences in English T1 & T6 (both LE prevalent)

GLMER analysis of screen difference in English T1		
	The number of fixations	
	null model	sides of screen
	(1)	(2)
Left		-0.001 (.962)
Right		−0.276 (<.001)
Constant	0.407 (<.001)	0.457 (<.001)
Random effects		
	Variance	*SD*
Participant (38)	0.0257	0.1603
Observations	6,168	6,168
Log likelihood	−8,252.810	−8,198.181
Akaike inf. crit.	16,513.620	16,408.360
Bayesian inf. crit.	16,540.530	16,448.720
GLMER analysis of screen difference in English T6		
	The number of fixations	
	Null model	Sides of screen
	(1)	(2)
Left		0.586 (<.001)
Right		−0.220 (<.001)
Constant	0.810 (<.001)	0.494 (<.001)
Random effects		
	Variance	*SD*
Participant (38)	0.0713	0.2671
Article (21)	0.0012	0.0357
Page (113)	0.0127	0.1128
Observations	7,586	7,586
Log likelihood	−13,332.040	−12,347.870
Akaike inf. crit.	26,672.080	24,707.740
Bayesian inf. crit.	26,699.810	24,749.340

*Note. p* value in brackets.

**Table 2:** GLMER analysis of screen differences in Chinese T1 & T6 (both LE prevalent)

GLMER analysis of screen difference in Chinese T1		
	The number of fixations	
	Null model	Sides of screen
	(1)	(2)
Left		0.122 (<.001)
Right		−0.195 (<.001)
Constant	0.423 (<.001)	0.409 (<.001)
Random effects		
	Variance	*SD*
Participant (36)	0.0408	0.2020
Article (21)	0.0022	0.0475
Page (103)	0.0082	0.0906
Observations	5,571	5,571
Log likelihood	−7,703.130	−7,638.904
Akaike inf. crit.	15,414.260	15,289.810
Bayesian inf. crit.	15,440.760	15,329.560
GLMER analysis of screen difference in Chinese T6		
	The number of fixations	
	Null model	Sides of screen
	(1)	(2)
Left		0.462 (<.001)
Right		−0.132 (<.001)
Constant	0.775 (<0.001)	0.561 (<.001)
Random effects		
	Variance	*SD*
Participant (36)	0.0526	0.2295
Article (21)	0.0069	0.0832
Page (103)	0.0180	0.1345
Observations	7,283	7,283
Log likelihood	−12,469.160	−11,906.030
Akaike inf. crit.	24,946.320	23,824.060
Bayesian inf. crit.	24,973.890	23,865.420

*Note. p* value in brackets.

Furthermore, Table 3 shows T2 and T3 (right-eye priority) having significantly more value on the right side of the screen in English readers, and T3 having significantly less value on the left side. Finally, Table 4 shows Chinese T2 and T3 have more value on the right side and significantly less value on the left side of the screen.

**Table 3:** GLMER analysis of screen differences in English T2 & T3 (both RE prevalent)

GLMER analysis of screen difference in English T2		
	The number of fixations	
	Null model	Sides of screen
	(1)	(2)
Left		0.023 (.485)
Right		0.117 (<.001)
Constant	0.312 (<.001)	0.249 (<.001)
Random effects		
	Variance	*SD*
Participant (38)	0.0134	0.1157
Observations	5,089	5,089
Log likelihood	−6,390.994	−6,381.292
Akaike inf. crit.	12,785.990	12,770.580
Bayesian inf. crit.	12,799.060	12,796.720
GLMER analysis of screen difference in English T3		
	The number of fixations	
	Null model	Sides of screen
	(1)	(2)
Left		−0.377 (<.001)
Right		0.027 (.0443)
Constant	0.924 (<.001)	1.013 (<.001)
Random effects		
	Variance	*SD*
Participant (38)	0.0682	0.2612
Article (21)	0.0010	0.0330
Page (113)	0.0210	0.1450
Observations	10,317	10,317
Log likelihood	−18,304.070	−17,903.600
Akaike inf. crit.	36,616.140	35,819.200
Bayesian inf. crit.	36,645.110	35,862.650

*Note. p* value in brackets.

**Table 4:** GLMER analysis of screen differences in Chinese T2 & T3 (both RE prevalent)

GLMER analysis of screen difference in Chinese T2		
	The number of fixations	
	Null model	Sides of screen
	(1)	(2)
Left		−0.071 (.0248)
Right		0.205 (<.001)
Constant	0.412 (<.001)	0.324 (<.001)
Random effects		
	Variance	*SD*
Participant (36)	0.0347	0.1864
Article (21)	0.0028	0.0530
Page (103)	0.0031	0.0561
Observations	5,153	5,153
Log likelihood	−7,074.346	−7,015.018
Akaike inf. crit.	14,156.690	14,042.040
Bayesian inf. crit.	14,182.880	14,081.320
GLMER analysis of screen difference in Chinese T3		
	The number of fixations	
	Null model	Sides of screen
	(1)	(2)
Left		−0.133 (<.001)
Right		0.125 (<.001)
Constant	0.899 (<.001)	0.887 (<.001)
Random effects		
	Variance	*SD*
Participant (36)	0.0728	0.2699
Article (21)	0.0028	0.0529
Page (103)	0.0283	0.1684
Observations	8,963	8,963
Log likelihood	−15,850.500	−15,722.270
Akaike inf. crit.	31,708.990	31,456.550
Bayesian inf. crit.	31,737.400	31,499.150

*Note. p* value in brackets.

This systematic binocular pattern is further seen in T5 (right-eye priority) and T7 (left-eye priority) in both orthographies, though the data are relatively sparse. We find significantly more value at the right side compared with the middle in both English and Chinese T5 (see Table 5). Results also show significantly more value at the left side compared with the middle in T7 in both orthographies (see Table 6). In addition, the results of English T4 (early-right and late-left priority) shows a significant concentration in the middle with significantly less at the left side (Est = −0.1269, *SE* = 0.0427), *z*(2700) = −2.972, *p* < .01, as well as the right side (Est = −0.1141, *SE =* 0.0422), *z*(2700) = −2.703, *p* < .01.

**Table 5:** GLMER analysis of screen differences in English & Chinese T5 (RE prevalent)

GLMER analysis of screen difference in English T5		
	The number of fixations	
	Null model	Sides of screen
	(1)	(2)
Left		−0.038 (.547)
Right		0.111 (.0164)
Constant	0.198 (<.001)	0.135 (<.001)
Observations	2,350	2,350
Log likelihood	−2,701.678	−2,696.226
Akaike inf. crit.	5,407.357	5,400.452
Bayesian inf. crit.	5,418.881	5,423.501
GLMER analysis of screen difference in Chinese T5		
	The number of fixations	
	Null model	Sides of screen
	(1)	(2)
Left		0.018 (.749)
Right		0.147 (.001)
Constant	0.243 (<.001)	0.153 (<.001)
Random effects		
	Variance	*SD*
Participant (36)	0.0037	0.0615
Observations	2,336	2,336
Log likelihood	−2,784.874	−2,777.907
Akaike inf. crit.	5,573.748	5,563.813
Bayesian inf. crit.	5,585.260	5,586.838

*Note. p* value in brackets.

All random intercepts were equal to zero.

**Table 6:** GLMER analysis of screen differences in English & Chinese T7 (LE prevalent)

GLMER analysis of screen difference in English T7		
	The number of fixations	
	Null model	Sides of screen
	(1)	(2)
Left		0.142 (.003)
Right		−0.071 (.370)
Constant	0.194 (<.001)	0.110 (.007)
Random effects		
	Variance	*SD*
Participant (38)	<.0001	<.0001
Observations	1,962	1,962
Log likelihood	−2,248.452	−2,241.014
Akaike inf. crit.	4,500.904	4,490.028
Bayesian inf. crit.	4,512.067	4,512.355
GLMER analysis of screen difference in Chinese T7		
	The number of fixations	
	Null model	Sides of screen
	(1)	(2)
Left		0.158 (.002)
Right		−0.050 (.505)
Constant	0.189 (<.001)	0.094 (.046)
Random effects		
	Variance	*SD*
Participant (36)	0.0039	0.0631
Observations	1,869	1,869
Log likelihood	−2,156.164	−2,148.010
Akaike inf. crit.	4,316.329	4,304.021
Bayesian inf. crit.	4,327.395	4,326.153

*Note. p* value in brackets.

## Discussion

Overall, we have found that the distribution of small asynchronies of binocular fixation accord with ocular prevalence, in line with our prediction. When readers fixate toward the left of a line (and the left of peripersonal space), the left eye tends to be prioritized in starting and ending fixations. The converse happens for the right eye. Statistical modeling supports the visualization of the data seen in Figs. 3 and 4. Again in line with our prediction, we found strikingly similar qualitative patterns between the Chinese and English readers. This potential role for ocular prevalence confirms the increasing importance that reading researchers have given to binocular coordination of eye movements over the past two decades.

What is the fundamental advantage of such delicate switching of processing between the eyes? Why should we expect to see it? The visual pathways obey the neuroanatomical principle of contralateral projection. Thus, the left eye itself projects more strongly to the right hemisphere and the right eye to the left hemisphere (as well as contralateral projection *within* each eye), reflecting (a) the exclusively contralateral monocular crescent of the temporal hemifield of each eye, (b) the biased crossed projection of nasal retinal ganglion cells which drive the contralateral ocular dominance columns in V1, and (c) the blind spot representation in the ipsilateral visual cortex (Toosy et al., 2001). We should therefore expect the fluid reprioritizing of the input from either eye as the reader’s gaze crosses the line, to facilitate visual and lexical processing (cf. Obregón & Shillcock, 2012).

Binocular fusion crucially facilitates visual processing (e.g., Blake & Fox, 1973; Jones & Lee, 1981; Ogle, 1964). Fusion is a process that occurs over time and may be affected by the nonconjugate fixational eye movements (drift, nystagmus, and microsaccades; Otero-Millan et al., 2014), by the existence of spatial disparity between the fixation points of the two eyes (Liversedge et al., 2006) and by the directional details of any such disparity (Kim et al., 2012). But fusion raises the question of which eye’s input should have priority for higher, conscious processing, because there are necessarily always differences between the two inputs, whether because of the different lines of sight or the different physical parameters of the two eyes. In this sense, the exigencies of ocular prevalence pervade visual processing.

Switching prevalence within the binocular fusion of successive fixations thus becomes a central issue. The default solution seems to be that prevalence is given to the nearest eye to the target in peripersonal space; thus, in reading, the left eye tends to be prioritized at the left end of the line, and the right eye at the right end of the line. We suggest that the physical constraints on the two eyes are intimately engaged in determining ocular prevalence. In the data we have explored, these physical constraints have involved the start and end time for the fixations of the two eyes. We suggest that these small timing differences assist and reflect ocular prevalence.

Both of the orthographies studied progress left-to-right. One way to understand the results is that in return sweeps from the end of one line to the beginning of the next, the left eye had tended to travel faster than the right eye and thus arrive earlier to start the fixation. Smaller, regressive (i.e., right-to-left) eye movements on the same line may be further candidates for this explanation; longer such regressions may provide more scope for a left eye priority, again tending to emphasize the beginning of the line. Such right-to-left movements involve the left eye’s lateral rectus muscle, associated with faster acceleration (Robinson, 1964); the left eye gets there first.

The lateral rectus muscle is critical for *abductive* saccades—moving away from the nose. Its relative strength over the musculature controlling *adductive* saccades (toward the nose) means that left-to-right movements across the line of text will tend to favor the abducting right eye, which will tend to arrive earlier to start the fixation, particularly for longer saccades necessarily tending to land more toward the end of the line. T3 (more numerous), T4, and T5 are the early-right-priority types. We suggest that an earlier start to a fixation constitutes a stimulus for ocular prevalence, meaning the right eye tends to assume priority in the conscious perception of the text as the reader moves from left to right across the screen. Types T6 (more numerous), T7, and T8 are the early-left-priority types; the picture is clearest in the more numerous T6. We suggest that early left-eye priority tends to reset the prevalence to the left eye at the beginning of the line.

The other aspect of asynchrony is late-priority, when one eye continues to fixate for longer: T2 (more numerous), T5, and T8 are the late-right priority types; T1 (more numerous), T4, and T7 are the late-left-priority types. We suggest that one eye continuing to fixate longer at the end of a fixation can also cue a switch in ocular prevalence or confirm an existing prevalence. In a fixation preceding a return sweep, an extended right-eye fixation may also reflect a faster abductive beginning to the saccade by the left eye.

The effects in Chinese readers are somewhat more systematic. For instance, Type T1 in English readers (see Table 4) only shows a significantly smaller distribution on the right, whereas in Chinese readers (see Table 5), T1 shows a significantly greater distribution on the left as well as a smaller one on the right. Similarly, the T2 distribution is significantly less on the left side compared with English readers. This greater systematicity in Chinese reading might reflect the fact that Chinese text fills each line with evenly spaced characters, effectively right-justifying the text, requiring readers to proceed further across each line, into right peripersonal space.

In total, the right-priority types (English 24.5%, Chinese 23.3%) outnumber the left-priority types (English 20.0%, Chinese 18.7%) chiefly due to the more numerous T3 types compared with T6 types. We suggest that this difference reflects the greater proportion of left-to-right (i.e., right eye abducting) saccades in the two languages.

Our data have concerned *temporal* differences in binocular coordination. How do they compare with *spatial* differences in the synchronization of the two eyes? Parker et al. (2019) review the research on spatial binocular coordination and present new data on the effects of the return sweep from the end of one line to the beginning of the next. They report larger spatial fixation disparities associated with these long return sweeps compared with shorter intraline regressive fixations. They associate the longer line-initial fixations with lack of a parafoveal preview as opposed to the need for greater convergence. We suggest that the accumulation of T6 (left priority) fixations toward the left of our texts reflect the mechanics of eye movements, including the return sweep, as opposed to any lack of preview. We might expect some effect of the fact that the English text has a ragged right edge, reflecting differences in word length, whereas Chinese approximates to a justified right edge, reflecting the small, regular width of Chinese characters. Figures 3 and 4 show qualitatively similar distributions for T6 in the two languages, but with a denser accumulation of T6 fixations at the left of the English text. In the English texts, the distance from the end of one line to the beginning of the next was necessarily slightly shorter on average than in the Chinese, and less predictable in length and angle. These issues remain topics for further research.

Regarding ongoing research, we might predict that right-to-left orthographies such as Arabic and Hebrew will show a reverse effect of the predominance of early-right-priority (T3) reported here, given that the left eye in these readers will be the abducting eye in most saccades. We should see more T6 types in those readers.

We may also predict that some dyslexics will show less exact control of the types of eye movements we have been considering (see, e.g., Raghuram et al., 2018). The fluid division of labor we have inferred between the eyes results in a fluctuating hemispheric division of labor. Various researchers have suggested a hemispheric dimension to dyslexia (e.g., Fabbro et al., 2001; Markee et al., 1996; Monaghan & Shillcock, 2008).

Finally, the data suggest two questions. First, the temporal asynchronies we have measured are small, typically of the order of a few milliseconds; these measurements necessarily depend on EyeLink II technology and its algorithm for calculating saccade onset and offset. Are the differences “artefactual” in some way? Understanding the process of saccade onset and offset and its empirical measurement is an ongoing research question (e.g., Bao, 2019; Hooge et al., 2015; Hooge et al., 2016; Hooge et al., 2019). For now, we report a predictable, interpretable pattern of temporal asynchronies arising from the mechanics of saccades as measured by EyeLink eye tracking. The study raises further issues that are particularly relevant to reading: What types of visual processing occur at what times within fixations and saccades? Is there differential processing of high and low spatial frequencies, for instance, or of different colors? How are these different aspects of visual processing related to ocular prevalence?

Second, are the small differences we have reported relevant to processing? We suggest that the potential importance of a timing difference in the afferent sensory apparatus is not comparable to the behavioral response times in laboratory psychology tasks, where small differences can be seen as inconsequential. Rather, we are dealing with a *computational* issue in which we are trying to understand a complex system based on the firing of very large numbers of neurons mediated by even larger numbers of synapses. The evidence is that spike-timing-dependent processing can play a key role in learning (cf. Hopfield & Brody, 2004). Small differences can have big effects. We propose that these asynchronies are informative enough to drive ocular prevalence, such that the input to one or other eye may be prioritized in the higher, binocularly-fused visual processing.

## Conclusions

We have analyzed small timing mismatches between the two eyes at the start and end of binocular fixations in English and Chinese reading. We argue that such asynchronies are predictive of ocular prevalence, in which input to the left eye is prioritized in conscious perception of a fused visual stimulus for targets in the left visual field and right-eye input is prioritized for targets in the right visual field. Ocular prevalence optimizes perception by (a) allowing the optimal interaction of monocular processing, respecting the differences in distortion and range for the images in the two eyes and their particularities, and (b) respecting the contralateral advantage. Having one eye’s fixation *begin* even slightly earlier may elicit prevalence for that input in higher perception and cognition. Having one eye’s fixation *end* even slightly later may also be a way of eliciting a switch in prevalence or of respecting the existing prevalence. The distribution of asynchronies is very similar across the two very different orthographies of Chinese and English, suggesting it may apply across all left-to-right orthographies. A picture emerges in which the physical constraints on binocular eye movements are intimately involved with the higher-level conscious processing of the fused image.

## Data Availability

None of the data for the experiments reported here is currently available, and none of the experiments was preregistered.

## Footnotes

1 The data were taken from the Edinburgh 5-Language Corpus, a large corpus of crosslinguistic binocular eye-tracking data assembled for the purpose of testing an open-ended number of hypotheses concerning reading behaviour. No formal power calculations were therefore performed, and there was no statistically motivated stopping criterion for testing.

2 The newspaper stories were all originally published in either English or Chinese (i.e., not translated) and only minimally edited to avoid dates and some proper names. The English stories were taken from *The Independent* newspaper; the Chinese newspaper stories were judged to be culturally equivalent by bilingual informants.
